# Accuracy of One Step malaria rapid diagnostic test (RDT) in detecting *Plasmodium falciparum* placental malaria infection in women living in Yaoundé, Cameroon

**DOI:** 10.1186/s12936-018-2595-8

**Published:** 2018-12-04

**Authors:** Rosette Megnekou, Jean Claude Djontu, Benderli C. Nana, Jude D. Bigoga, Maurice Fotso, Balotin Fogang, Rose F. G. Leke

**Affiliations:** 1 Department of Animals Biology and Physiology, Faculty of Sciences, University of Yaoundé I, PO. BOX: 812, Yaoundé, Cameroon; 2 The Biotechnology Center, University of Yaoundé I, P.O. Box 3851, Messa, Yaoundé, Cameroon; 3 Department of Biochemistry, Faculty of Sciences, University of Yaoundé I, P.O. Box 812, Yaoundé, Cameroon; 4 School of Health Science, Catholic University of Central Africa, P.O. Box 1110, Yaoundé, Cameroon

**Keywords:** *Plasmodium falciparum*, Placental tissue, Peripheral blood, RDT, Pregnant women, Cameroon

## Abstract

**Background:**

*Plasmodium falciparum* infected erythrocytes sequestering in placental tissue release *Plasmodium* lactate dehydrogenase (pLDH) and histidine-rich protein-II (HRP-II). These proteins can be detected in peripheral blood using monoclonal antibody-based rapid diagnostic tests (RDTs). Nevertheless, studies to evaluate the reliability of RDTs in detecting placental malaria compared with microscopy of placental tissue impression smear (PTIS) as the gold standard are scarce.

**Methods:**

Between August 2013 and January 2015, Giemsa-stained blood smears for peripheral blood smear (Pbs), placental intervillous space (IVS) blood smear and placental tissue impression smear (PTIS)] were prepared from HIV-negative women during delivery at the Marie Reine Medical Health Centre in Yaoundé, Cameroon. RDTs with monoclonal antibodies specific to HRP-II (P.f) or pLDH (Pan) antigens were used to screen maternal peripheral blood samples.

**Results:**

The prevalence of malaria was 16%, 7.5%, 11.5%, 8% and 13% for One Step malaria HRP-II and pLDH RDTs, peripheral blood smear, IVS blood and placental tissue impression smears, respectively. The proportion of women positive by One Step malaria pLDH RDT and Pbs increased with parasite density in PTIS, while One Step malaria HRP-II RDT detected high proportion of infected women even with low parasite density. Although the prevalence of malaria infection by both microscopy and RDTs decreased significantly with mother age (0.0008 ≤ p ≤ 0.025), parity seemed to have very little influence. The sensitivity of One Step malaria HRP-II and pLDH RDTs were 96.15% and 61.53%, respectively, compared to 80.76% for Pbs (p = 0.014 and 0.0029, respectively). The specificity of these RDTs was 96.49% and 100%, respectively, compared to 100% for Pbs (p ≥ 0.12). In addition, the positive predictive values were 80.64% and 100% for HRP-II and pLDH-based RDTs, respectively, compared to 100% for Pbs (p < 0.0001 and 1, respectively), while the negative predictive values were 99.40% and 94.48%, respectively, compared to 97.16% for Pbs (p ≥ 0.49). The combination of One Step malaria HRP-II RDT and Pbs showed the similar performance as that observed with One Step malaria HRP-II RDT only.

**Conclusion:**

These results depict One Step malaria HRP-II RDT to be better in detecting placental *P. falciparum* infection in pregnant women compared to Giemsa-stained peripheral thick blood smear. This is important for better case management since microscopic examination of PTIS cannot be employed during pregnancy.

## Background

Malaria remains one of the most important parasitic infections in humans. It is endemic throughout the tropical and subtropical regions of the world and is responsible for more than 200 million clinical cases and more than 400 thousand deaths each year [1]. Most of these deaths occurred in the sub-Saharan African (92%), the South-East Asian (6%) and the Eastern Mediterranean Regions (2%) [1]. Children of less than five years of age and pregnant women are the most susceptible. In pregnant women, *Plasmodium falciparum*-infected erythrocytes (IE) express a VAR2CSA antigen that mediates binding of IE to the chondroitin sulfate A (CSA) on syncytiotrophoblasts lining the intervillous space (IVS) of the placenta [2]. In fact, the World Health Organization (WHO) considers malaria in pregnancy as one of the most important causes of low birth weight deliveries worldwide and a major cause of severe maternal anaemia contributing to maternal mortality. In addition, placental malaria is the most important contributing factor to premature delivery, hypertension, infant anaemia and neonatal mortality [3].

In recent years, there has been a rapid scale up of interventions, including the use of long-lasting insecticide-treated nets (LLINs), indoor residual spraying, intermittent preventive treatment, seasonal malaria chemoprevention and treatment with artemisinin-based combination therapy (ACT) [1]. Nevertheless, malaria still remains a major health problem in sub-Saharan Africa because vectors are evolving and developing insecticide resistance and antimalarial drug resistance may be a potential risk in the future [1]. Therefore, prompt and appropriate treatment of acute malaria is critical not only in preventing progression to severe disease and death, but also in halting the evolution and spread of resistance to currently used anti-malarial drugs. Efficient treatment is guided by correct diagnosis of the disease. In fact, accurate diagnosis of malaria might improve the management of febrile illnesses and ensure anti-malarial medicines be used only when necessary. Thus, any malaria elimination programme should include methods for accurate and sensitive detection of *Plasmodium* infection.

Although the WHO considers microscopy as the standard method for the detection of malaria parasites in humans [4], about 20% of Cameroonian women at delivery with malaria positive placental tissue impression smear have been shown to present negative peripheral and intervillous space thick blood smear [5]. Molecular techniques were shown to be highly sensitive, with PCR detecting as low as 1–5 parasites/µL of blood [6, 7]. However, such techniques are expensive, need highly trained technicians and are not advantageous in areas of high malaria transmission where submicroscopic parasitaemia is prevalent in human and may be important for maintaining natural immunity to malaria parasites. Rapid diagnostic tests (RDTs) may be a potential alternative since a strong association exists between prevalence of malaria by this technique and prevalence by microscopy [8], among non-pregnant population. Intermittent preventive treatment in pregnancy (IPTp) was implemented in Cameroonian women in 2005 and the current coverage rate is approximately 70% in urban settings [9]. Sulfadoxine–pyrimethamine used in IPTp acts by inhibiting the folic acid synthesis in malaria parasite, which is required for its replication. This treatment might decrease malaria parasitaemia in pregnant women, which could affect the accuracy of RDTs by driving parasitaemia below the test’s limit of detection. However, few published data on the performance of RDTs for diagnosing placental malaria are available, especially in Cameroonian pregnant women after IPTp implementation, and none of them used microscopic examination of placental tissue impression smear as gold standard. *Plasmodium falciparum* may be present in placenta tissue yet not detectable in peripheral and IVS blood smears by routine light microscopy [3, 10]. Although both histological and microscopic examinations of placental impression smears allow for the detection of parasite lodged in the small sinuses located among placenta villi (i.e. in the site where they are sequestered), the histological method has been shown to be less sensitive [11] and cannot be safely used to detect malaria infection during pregnancy. Therefore, microscopic examination of placental impression smears in this study is considered as the gold standard for diagnosing placental malaria. The rational of RDTs to efficiently diagnose placental malaria infection is that their targets antigens which are *Plasmodium* lactate dehydrogenase (pLDH) and histidine-rich protein-II (HRP-II), are released by *P. falciparum* infected erythrocytes sequestered in the intervillous space of the placenta tissue and can be detected in the peripheral blood [12]. Nevertheless, RDTs continue to show false positive results due to residual pLDH or HRP-II from 1 to 2 weeks following parasite clearance [6, 13, 14].

The present study sought to evaluate the accuracy of HRP-II and pLDH based malaria RDTs in the detection of placental malaria parasitaemia in women living in Yaoundé, Cameroon, after IPTp implementation compared to microscopic examination. The results of this study may help to identify a useful tool for better detection of placental *P. falciparum* infection in women during pregnancy.

## Methods

### Ethical considerations

The National Ethics Committee of Cameroon approved the study protocol (No. 029/L/CNERSH/SP). Administrative Authorizations were obtained from the Ministry of the Public Health of Cameroon (No. D30-392 AAR/MINSANTE/SG/DROS/CRC/CEA1) and from the Health Centre. Participation in the study was voluntary with written Informed Consent from each adult woman prior to enrollment. Parental assent was sought for participants less than 18 years old. The study was performed following the guidelines on human clinical research as recommended by the Ministry of the Public Health of Cameroon and data treated in the strict respect of anonymity.

### Study site and population

The study was cross sectional and took place from August 2013 to January 2015 at the Marie Reine Health Center in Etoudi, a peri-urban area of Yaoundé, Cameroon. Malaria transmission in this area is intense and perennial, peaking in the months of May (during the long-wet season from March to June) and October (during the short-wet season from September to November). A total of 197 HIV negative women aged 16 to 39 years, were recruited. Among these, 40% were from sub-urban areas (where malaria transmission is unknown), and 60% from urban settings where malaria transmission is low (about 13 infectious bites/person/year [15]. Information on the mother’s health, estimated length of pregnancy, parity, age, use of anti-malarial drugs and mosquito net were recorded in a questionnaire. Peripheral and IVS blood samples collected from women immediately following delivery were used for smear preparation required for malaria diagnostic by microscopy. RDTs and determination of haemoglobin level were performed using peripheral blood. Placental tissue was also collected as reported previously [5] and a section used to prepare impression smears. The characteristics of the study participants are summarized in Table 1.

**Table 1:** Characteristics of the study population

Parameters	Women at delivery (n = 197)
Median age, years	26 (16–39)
Primipara	71 (36%)
Secundipara	43 (22%)
Multipara	83 (42%)
Hb levels (g/dL)	12.2 (7.5–15.7)
Median parasitaemia in peripheral thick blood smear	4463 (167.5–273,870) parasites/µL
Median parasitaemia in placental intervillous thick blood smear	1306 (83.5–42,405) parasites/µL
Median parasite density in placental tissue impression smear	2.1% (0.006–93) %
Anaemic women	38 (19%)
Uptake of IPT-SP	162 (82%)
Use of insecticide treated bed nets (ITNs)	138 (70%)

Values in the parentheses represent either percentage or range (lowest and highest values)

*Hb* haemoglobin, *IPT-SP* intermittent preventive treatment using sulfadoxine-pyrimethamine

### Diagnosis of malaria infection by microscopy and determination of haemoglobin levels

In order to detect parasites in the peripheral and placenta, thick and thin blood smears were prepared using maternal peripheral and IVS bloods. In addition, impression smears were also made from placenta tissue. In brief, a small piece of placenta tissue was blotted on filter paper, then pressed three to five time on against a glace slide. After drying, all slides were stained by Giemsa and examined by two microscopists for the presence of malaria parasites and species determination. If parasites were not detected after examining 200 microscopic fields of thick smears, the sample was considered malaria negative. However, when parasites were detected, parasitaemia was estimated per microliter of blood. Both thin film and impression smear were used to determine parasite species. Impression smear was also used to estimate parasite density as percentage of infected erythrocytes per 20,000 red blood cells (RBC). In addition, One Step malaria RDTs with antibodies specific to HRP-II and pLDH were used to assess all maternal peripheral blood. The RDTs were stored between 4 and 30 °C according to manufacturer’s instructions. All microscopists were blinded to the RDT results. Haemoglobin (Hb) levels in maternal blood were determined using Coulter Counter (URIT-3300, Europe). A woman was anaemic if Hb < 11 g/dL.

### Malaria parasites diagnosis by RDT

Commercialized One Step^®^ HRP-II and pLDH RDT (SD Bioline malaria antigen P.f/pan, Standard Diagnostics Inc, Kyonggi-do, Korea) was used to diagnose malaria infection in the peripheral blood samples obtained from women following manufacturer’s instructions. Briefly, five microlitres (5 µL) of anticoagulated blood was applied to the RDT cassette then two drops of the buffer added to form a blood-buffer mixture and the result read within 10–15 min, and immediately recorded. The tests were interpreted as follows: (i) the appearance of only one coloured (red) band on the control window indicated a negative result; (ii) the appearance of two coloured (red) bands, one in the test window on the *P. falciparum* line and the other in the control window indicated *P. falciparum* infection only; (iii) when two coloured (red) bands were observed, one on the test window on the ‘pan’ line in addition to the control line, the test was positive to non-*P. falciparum* species (this was not observed in the present study); (iv) if there were three red coloured bands (two in the test window representing both the *P. falciparum* and pan lines in addition to the control line), the test was declared as mixed infection of *P. falciparum* with other *Plasmodium* species. The non-appearance of a red coloured line in the control window of the cassette indicates that the test is invalid. All invalid tests were repeated on a new cassette to ascertain the result.

### Statistical analysis

The Graph Pad Prism 5.03 and SigmaStat software were used for statistical analyses. Sensitivity, specificity, positive predictive value, and negative predictive value were used as standard parameters to assess the diagnostic performance of RDT in comparison with the microscopic examination of the placental tissue impression smear considered as the gold standard. Results were reported as proportion or median with 95% confidence intervals. Proportions were compared using Fisher’s exact test (two proportions) or χ^2^ test (more than two proportions). Multi-variate logistic regression analysis was developed to assess the association between the presence of parasite density in the placenta tissue and detection frequency of the infection using RDT, with adjustment for parity, mother’s age, IPTp and LLIN usage. The odds ratio with 95% confidence intervals were reported and p-values less than 0.05 were considered statistically significant.

## Results

### Description of the study population

A total of 197 HIV-negative women were enrolled into the study amongst which 36% [29.3–42.7%] were primipara, 22% [16.22–27.78%] secondipara and 42% [35.11–48.89%] multipara. The age ranged from 16 to 39 with a median age of 26. The median parasitaemia from peripheral and intervellous space thick blood smear was 4463 and 1306 parasites/µL respectively, while the median percentage of infected red blood cells in placental tissue impression smear was 2.1%. Concerning anaemia, 19% [13.52–24.48%] of women were anaemic. In this study population, 82% [76.64–87.36%] took intermittent preventive treatment in pregnancy-based sulfadoxine-pyrimethamine (IPTp-SP) against malaria and 70% [63.60–76.40%] slept under LLINs during pregnancy (Table 1). *Plasmodium falciparum* was the only *Plasmodium* species found.

### Prevalence of malaria infection by microscopy and RDT in women at delivery

**Fig. 1:**
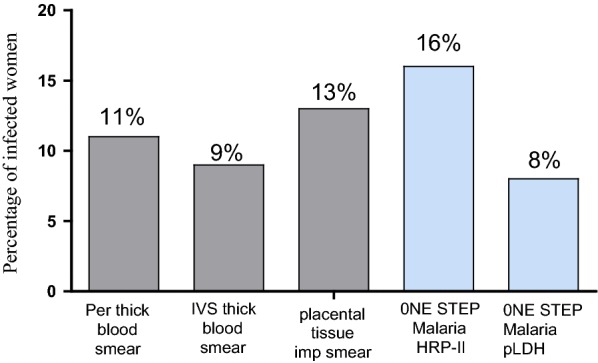
Prevalence of malaria infection by microscopy and RDT at delivery in women living in Yaoundé, Cameroon. *Per* peripheral blood, *IVS* placental intervillous space blood, *imp* impression

The prevalence of malaria infection was higher with One Step malaria HRP-II RDT and was 16% [10.88 - 21.12%] compared to that of: (i) One Step malaria pLDH RDT which was 8% [4.21–11.79%] (p = 0.12), (ii) the microscopic examination of placental tissue impression smear which was 13% [8.30–17.70%] (p = 0.68), (iii) the thick smear of peripheral blood which was 11% [6.63–15.37%] (p = 0.54) and, (iv) the thick smears of IVS blood which was 9% [5.00–13.00%] (p = 0.12) (Fig. 1), although the differences were not statistically significant.

### Variation of the infection rate in peripheral blood of women at delivery as function of parasite density in the placental tissue impression smear

**Fig. 2:**
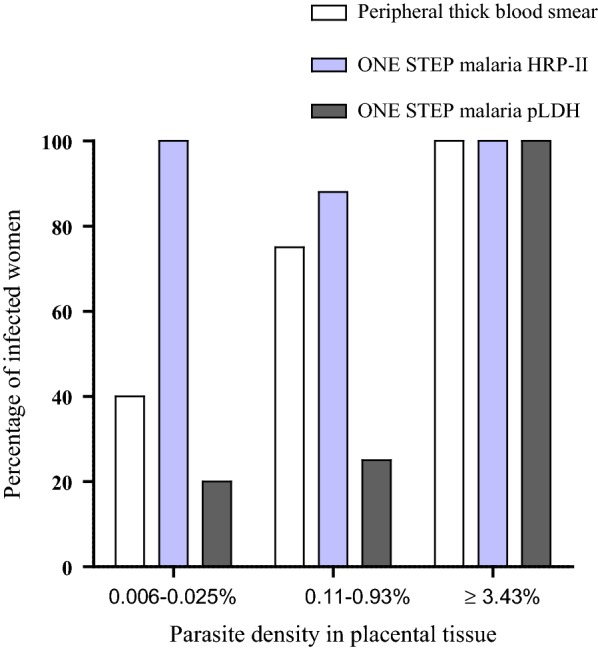
Proportion of women with malaria positive by RDT and peripheral thick blood smear as function of the parasite density (% infected red cells) in placental tissue impression smears

In order to investigate whether parasite density in the placenta tissue had an influence on infection detection by microscopy and RDTs in the peripheral blood, the proportion of women with positive thick smear and RDTs performed with peripheral blood was analysed in relation to parasite density in the placental tissue impression smear. As shown in Fig. 2, when, the parasite density ranged between 0.006 and 0.025%, the proportion of positive results by peripheral thick blood smear, One Step^®^ malaria HRP-II and One Step malaria pLDH RDT was 40% [33.16–46.84%], 100% and 20% [14.41–25.59%], respectively. For parasite densities ranging between 0.11 and 0.93%, the corresponding values for the different methods were 78% [72.22–83.78%], 85% [80.01–89.99%] and 22% [16.22–27.78%], respectively. When parasite density was greater than or equal to 3.43%, the infection rate was 100% with all three diagnostic methods. Using Chi Square test, results showed a significant association of placental parasite density with malaria detection by peripheral thick blood smear (χ^2^ = 8.53 and p = 0.0035) and One Step malaria pLDH RDT (χ^2^ = 13.62 and p = 0.0002), but not with One Step malaria HRP-II (χ^2^ = 0.165 and p = 0.68).

### Prevalence of malaria infection of women at delivery in relation with parity and mother age

**Fig. 3:**
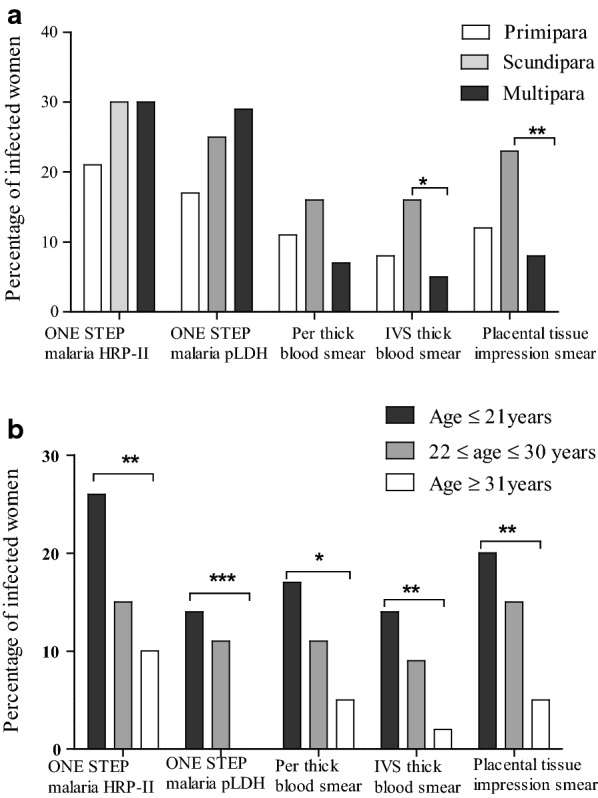
Prevalence of malaria infection in relation with parity (**a**) and age (**b**) according to different diagnostic methods. *Per* peripheral blood, *IVS* placental intervillous space blood, *imp* impression. *p < 0.05; **p < 0.009; ***p < 0.0009

Although no significant association was found in this study between malaria infection by RDT and parity, the infection frequency by microscopic of IVS blood and placental tissue impression smear was lower in multipara and was 5% [0.31–9.69%] and 8% [2.16–13.84%], respectively) compared to secondipara which was 16% [5.00–26.00%] and 23% [1210.42–35.58%] (p = 0.019 and 0.0035, respectively) and primipara women which was 8% [1.68–14.31%] and 12% [4.44–19.56%] (p = 0.40 and 0.39, respectively) (Fig. 3a). The prevalence of malaria infection by both RDT and microscopy was significantly higher in women less than or equal to 21 years compared to those aged 22–30 years and those aged greater than or equal to 31 years (p = 0.0087, 0.0008, 0.025, 0.0086 and 0.0064 for One Step malaria HRP-II, One Step malaria pLDH, peripheral thick blood smear, IVS thick blood smear and placental tissue impression smear, respectively) (Fig. 3b). These suggest that mother’s age might influence susceptibility to pregnancy-associated malaria more than parity since maternal immunity to malaria might increase with age.

Since mother’s age and parity are associated with malaria infection or risk of infection by RDTs in uni-variate analysis, the impact of these variables was jointly tested with parasite density, IPTp and bed net usage in multi-variate logistic regression model. Increasing parasite density in the placenta tissue increased the odds of infection detection by HRP-II based RDT (OR = 8.9E+014; CI 1,272,716–6E+023; p < 0.001), but not by that of pLDH based RDT (OR > 1e40; CI 8.7E−055–> 1e40; p = 0.33) after adjusting for parity, mother age, IPTp and bed net usage (Table 2).

**Table 2:** Odds ratio for the association between parasite density in placental tissue and the detection of malaria infection by RDTs

	Positive HRP-II based RDT	Positive pLDH based RDT
Density of infected red blood cells in the placental tissue	8.9 E+014 [1,272,716–6E+023] p < 0.001	1e40 [8.7 E-055−1e40] p = 0.33
Parity	1.76 [0.93–3.32] p = 0.08	197.5 [0.03–1,315,700] p = 0.24
Mother age	0.87 [0.72–1.05] p = 0.15	0.10 [0.002–3.85] p = 0.25
IPTp usage	0.76 [0.46–1.34] p = 0.36	1.68E−014 [1.24E−045–2.30E+017] p = 0.38
LLINs usage	1.28 [0.55–6.50] p = 0.75	0.00 [1.82 E−012−4951.53] p = 0.31

ORs with 95% CIs and p-values reported based on multi-variate logistic regression

### Accuracy of microscopic examination of Giemsa-stained peripheral thick blood smear and RDT in comparison with Giemsa-stained placental tissue impression smear in women at delivery

**Fig. 4:**
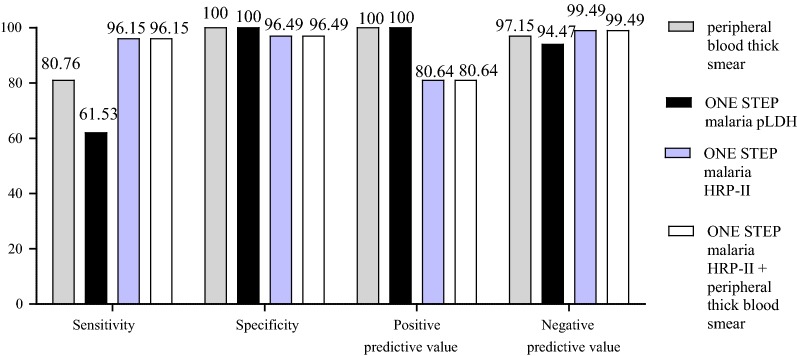
Accuracy of microscopic examination of Giemsa-stained peripheral thick blood smear and RDT in comparison with Giemsa-stained placental tissue impression smear in women at delivery

In order to determine the accuracy of RDT used, the results were analysed taking into consideration peripheral thick blood smear, One Step malaria HRP-II and One Step malaria pLDH RDTs, each alone, and One Step malaria HRP-II plus peripheral thick blood smears together, using placental impression smear as the gold standard (Table 3 and Fig. 4). As observed, the sensitivity, specificity, positive predictive value (PPV) and negative predictive value (NPV) for microscopic examination of Giemsa-stained peripheral thick blood smear were 80.77% [60.65–93.45%], 100% [97.87–100%], 100% [83.89–100%] and 97.16% [93.50–99.07%], respectively. The corresponding values for One Step malaria HRP-II and One Step malaria pLDH RDTs were 96.15% [80.36–99.90%], 96.49% [92.52–98.78%], 80.65% [62.53–92.55%], 99.40% [96.69–99.98%] and 61.54% [40.57–79.77%], 100% [97.87–100%], 100% [79.41–100%], 94.48% [90.07–97.32%], respectively. The combination of microscopic examination of Giemsa-stained peripheral thick blood smear and One Step malaria HRP-II RDT showed a similar result as that of One Step malaria HRP-II (Fig. 4 and Table 3).

**Table 3:** Accuracy of RDT and peripheral thick blood smear in comparison with placental tissue impression smear for detecting placental malaria infection in 197 women at delivery

	Placental tissue impression smear			Accuracy measure in % (95% CI)	
	Positive	Negative	Total		
Per thick blood smear					
Positive	21	0	21	Sensitivity	80.77 (60.65–93.45)
Negative	5	171	176	Specificity	100 (97.87–100)
Total	26	171	197	Positive predictive value	100 (83.89–100)
				Negative predictive value	97.16 (93.50–99.07)
One Step malaria pLDH					
Positive	16	0	16	Sensitivity	61.54 (40.57–79.77)
Negative	10	171	181	Specificity	100 (97.87–100)
Total	26	171	197	Positive predictive value	100 (79.41–100)
				Negative predictive value	94.48 (90.07–97.32)
One Step malaria HRP-II					
Positive	25	6	31	Sensitivity	96.15 (80.36–99.90)
Negative	1	165	166	Specificity	96.49 (92.52–98.78)
Total	26	171	197	Positive predictive value	80.65 (62.53–92.55)
				Negative predictive value	99.40 (96.69–99.98)
One Step malaria HRP-II + peripheral thick blood smear					
Positive	25	6	31	Sensitivity	96.15 (80.36–99.90)
Negative	1	165	166	Specificity	96.49 (92.52–98.78)
Total	26	171	197	Positive predictive value	80.65 (62.53–92.55)
				Negative predictive value	99.40 (96.69–99.98)

The comparison shows that the sensitivity of One Step malaria HRP-II RDT was significantly higher than that of peripheral blood smear [current diagnostic method (p = 0.0014)] while the specificity was not statistically different between the two tests (p = 0.12). In addition, no significant difference was observed with NPV between the two tests while PPV was higher with peripheral blood smear (p < 0.0001). Peripheral blood smear and HRP-II based RDT were both more sensitive than pLDH based RDT (p = 0.0029 and p < 0.0001, respectively). No significant difference was observed with specificity, PPV and NPV between these tests.

### Accuracy of microscopic examination of Giemsa-stained peripheral thick blood smear and RDT in comparison with Giemsa-stained placental tissue impression smears in women by parity status

The susceptibility to pregnancy-associated malaria has been shown to increase in primipara women as well as with parasite density. Therefore, an investigation was performed in order to determine whether parity could influence the performance of RDTs in the diagnosis of placental malaria. Although the results showed significant variations in some parameters with RDTs performance between primipara, secundipara and multipara women (Table 4), these variations seem not to be specific for parity and need to be investigated further.

**Table 4:** Accuracy of RDTs and peripheral thick blood smear in comparison with placental tissue impression smear for detecting placental malaria infection according to the parity

	Primi	Secundi	Multi	p–value
Pbs				
Sensitivity (%)	88.88 [51.75–99.72]	70.0 [34.75–93.33]	85.7 [42.13–99.64]	0.009
Specificity (%)	100 [94.22–100]	100 [89.42–100]	100 [95.26–100]	–
PPV (%)	100 [63.06–100]	100 [59.04–100]	100 [54.07–100]	–
NPV (%)	98.40 [91.47–99.96]	91.66 [77.53–98.25]	98.79 [92.98–100]	0.19
pLDH based RDT				
Sensitivity (%)	55.55 [21.20–86.30]	60.00 [26.24–87.84]	71.43 [29.04–96.33]	0.05
Specificity (%)	100 [94.22–1.00]	100 [89.42–100]	100 [95.26–100]	–
PPV (%)	100 [47.82–1.00]	100 [54.07–100]	100 [47.82–100]	–
NPV (%)	93.10 [85.20–98.32]	89.19 [74.58–96.97]	98.27 [91.04–99.69]	0.03
HRP-II based RDT				
Sensitivity (%)	88.88 [51.75–99.72]	100 [69.15–100]	100 [59.04–100]	< 0.0001
Specificity (%)	96.77 [88.83–99.61]	100 [89.42–100]	94.74 [87.07–98.55]	0.05
PPV (%)	80.00 [44.39–97.48]	100 [69.15–100]	63.64 [30.79–89.07]	< 0.0001
NPV (%)	98.21 [91.20–99.96]	100 [89.42–100]	100 [95.01–100]	0.13
HRP-II based RDT + Pbs				
Sensitivity (%)	88.88 [51.75–99.72]	100 [69.15–100]	100 [59.04–100]	< 0.0001
Specificity (%)	96.77 [88.83–99.61]	100 [89.42–100]	94.74 [87.07–98.55]	0.05
PPV (%)	80.00 [44.39–97.48]	100 [69.15–100]	63.64 [30.79–89.07]	< 0.0001
NPV (%)	98.21 [91.20–99.96]	100 [89.42–100]	100 [95.01–100]	0.13

Values in square brackets represent 95% confidence intervals. Chi Square test was used to compare the performance of different malaria diagnostic methods between primipara, secundipara and multipara women

*Primi* primipara women, *Secundi* secundipara women, *Multi* multipara women, *PPV* positive predictive value, *NPV* negative predictive value, *Pbs* peripheral blood smear

## Discussion

The diagnosis of placental malaria in women during pregnancy remains a major challenge. Although the WHO considers microscopic examination of Giemsa-stained peripheral, thick blood smears as the standard method for the detection of malaria parasite in humans [4], about 20% of Cameroonian women at delivery with malaria positive placental tissue impression smear were reported to present negative peripheral thick blood smear [5]. This high proportion of undiagnosed women infected with malaria in pregnancy constitutes one of the major difficulties in the fight for malaria elimination. Therefore, the search for better alternatives to microscopy for placental malaria diagnosis in women such as RDT or others appears imperative with the vision of Global Technical Strategy to have a malaria free world by 2030. The prevalence of placental malaria in the present study was 13%. This prevalence is lower than that (22%) reported in the same setting in 2005 [16], but remains worrisome. This decline of malaria incidence in pregnant women living in Yaoundé, Cameroon reflects the general decrease of malaria prevalence observed across the world throughout this last decade [1].

In general, the prevalence of malaria infection in the present study was higher with One Step malaria HRP-II RDT (16%) than with One Step malaria pLDH RDT (7.5%), microscopic examination of placental tissue impression smear (13%), thick smear of peripheral blood (11.5%) and thick smear of placental tissue intervillous space blood (IVS) (8%). In addition, the percentage of malaria positive cases with placental tissue impression smear was higher in the present study compared to that of peripheral blood thick smear. This result is similar to the findings of previous studies [5, 11]. The possible explanation may be the ability that *P. falciparum* infected erythrocytes has to sequester in the placental tissue through expression of a VAR2CSA antigen, which mediates their binding to the chondroitin sulfate A (CSA) on syncytiotrophoblasts lining the intervellous space of the placenta [2].

The high prevalence of malaria infection observed with One Step malaria HRP-II RDT suggest that it can detect great proportion of malaria positive cases undetected by peripheral thick blood smears. In fact, of the five cases of malaria positive placental tissue impression smear with negative peripheral thick blood smears observed in the present study, four were positive to HRP-II based RDT. It is known that *P. falciparum* can be present in the placenta tissue, but not detectable in the peripheral blood [3, 10]. Thus, the one case detected in this study with impression smear and not with HRP-II based RDT could be due to the very low parasite density in the placenta tissue. Nevertheless, false positive results have been reported with HRP-II based RDT and can be explained by the fact that this *P. falciparum* antigen can persist in circulation for about 2 weeks following parasite clearance [6, 12]. Conversely, the low prevalence observed by One Step malaria pLDH RDT is suggestive of low sensitivity, but increased specificity compared to the HRP-II test.

In the present study, the relationship between malaria prevalence with parity and mother age was examined. Unlike in previous studies showing significant associations between increased parity and decreasing susceptibility to pregnancy-associated malaria [11, 16], the cohort in this study did not show a significant difference between parity and malaria infection. However, the malaria infection was significantly more prevalent in women less than 21 years old than in those older. The decrease susceptibility to placenta malaria infection with increasing mother age corroborates the findings of Meghna et al. [17] and supports the idea of possible existence of maternal age-dependent immunity that can be important for the protection against malaria infection in pregnant women living in stable malaria transmission setting. Also, the proportion of placental malaria positive women with negative peripheral thick blood smears was 19.23% (5/26), similar to other previous studies that reported ranges from 19 to 22% [5, 11]. This result indicates that a high proportion of pregnant women with placental malaria infection are left untreated since the microscopic examination of Giemsa-stained peripheral thick blood smear is the routine diagnostic method used in Cameroon and across Africa. In fact, in high transmission intensity, where levels of acquired immunity tend to be high, *P. falciparum* infection is usually asymptomatic in pregnancy. Yet parasites may be present in the placenta and contribute to maternal anaemia even in the absence of documented peripheral parasitaemia [18]. Both maternal anaemia and placental parasitaemia can lead to low birth weight, which is an important contributor to infant mortality. The proportion of women with positive One Step malaria pLDH RDT was found to increase with increasing parasite density in placental tissue impression smears. However, One Step malaria HRP-II RDT detected high proportion of infected women even with low parasite density in the placenta tissue. Previous study from Cameroon reported that the percentage of HRP-II ELISA positive results from peripheral plasma of women correlated with placental parasitaemia [5].

These observations justify the necessity of several studies carried out in different eco-epidemiological zones for malaria to determine the context or condition in which RDTs will be efficient for diagnosing pregnancy-associated malaria. Among previous studies that assessed the performance of RDTs for the diagnosis of placental malaria, only the study by Leke et al. used placental impression smears as the standard [5] technique, having been shown to be more sensitive than the histological examination of placental tissue [19]. However, their study was carried out in 1999 before the implementation of IPT in pregnant women. The sulfadoxine-pyrimethamine used in IPTp acts by inhibiting the folic acid synthesis, which is required for malaria parasite replication, thus might decrease malaria parasitaemia in pregnant women. The previous observation that the detection of malaria infection by RDTs increases with parasitaemia [20], justify the necessity of the present study carried out few years after IPTp implementation in Cameroon.

Concerning sensitivity and specificity when using placental impression tissue smears as gold standard, the sensitivity of One Step HRP-II was higher and that of pLDH RDT lower compared to that of peripheral blood smears while the specificity of RDTs ranged between 96 and 100% compared to 100% for peripheral blood smears. Concerning the positive and negative predictive values, while the PPV for RDTS ranged between 80.64 and 100% compared to 80.76% for peripheral blood smears; the NPV of the RDTs ranged between 99.39 and 94.4% compared to 97.15% for peripheral blood smears. These results demonstrate that the performance of HRP-II based RDT used in this study for placental malaria diagnosis was in general, higher compared to previous study from Cameroon [5] and other countries in sub-Saharan Africa [21–23]. One of the explanations of the difference reported in RDT performance between these different studies is the fact that unlike to the present one, which used placental impression smears as the standard; other studies used histological examination of placental tissue, shown to be less sensitive. Another reason could be the manufacturer condition of antigens that can change slightly according to company. In addition, the eco-epidemiological zones for malaria have an impact on the parasite density that can influence the detection of malaria infection using RDTs [24]. The very low percentage of false negative results (0.6%, 1/166) observed in this study with HRP-II based RDT is desirable and indicates the high sensitivity of the test. These false negative results could be due to low parasite density as has been reported elsewhere [25, 26]. Results also showed that, the percentage of false positive results was 19.36 (6/31) and this could be due to the ability of RDT to pick up remnant parasite antigens in patients who were possibly on treatment and whose parasitaemia was cleared. These false positives associated with the HRP-II based RDT represent a potential limitation of the test. On the other hand, the high sensitivity (96.5%) of One Step malaria RDT may be useful for countries moving towards elimination to better detect asymptomatic and submicroscopic infections. In general, the findings of this study validate the fact that HRP-II based RDT may be more accurate in the detection of placental *P. falciparum* parasitaemia in pregnant women compared to Giemsa-stained peripheral thick blood smear.

To be recommended by the WHO, any RDT need to have a sensitivity and specificity beyond 95% compared to standard method. With a sensitivity of 96.5% and specificity of 96.1% One Step malaria HRP-II based RDTs may be an important tool for detecting placental *P. falciparum* infection in women during pregnancy. This suggestion is supported by the fact that the combination of One Step malaria HRP-II RDT and peripheral blood smears showed in this study the similar performance as that observed with One Step malaria HRP-II RDT. Although One Step malaria pLDH RDT showed a good specificity and positive predictive value, its sensitivity was very poor suggesting that this RDT cannot be used alone to accurately diagnose placental malaria. The enzyme pLDH is released from viable parasitized blood cells and is rapidly detected by series of monoclonal antibodies. Since pLDH is the product of viable parasites, the test may be used to monitor effective antimalarial therapy due to the very short half-life in circulation following parasite clearance compared to HRP-II or that negative results can be confirmed with an additional HRP-II based test.

A previous study reported high sensitivity of HRP-II based RDT in multigravida and secundigravida women compared to primigravida women [23], suggesting that parity may affect RDTs performances in the diagnosis of placental malaria. Although results from this study showed significant variations in some parameters in RDT performance between primipara, secundipara and multipara women, these variations seem not to be specific to parity, suggesting the need for further investigations.

## Conclusions

These results indicate that HRP-II based RDT may be more accurate in detecting placental *P. falciparum* parasitaemia in pregnant women compared to Giemsa-stained peripheral thick blood smear. With a sensitivity and specificity higher than those recommended by WHO for the validation of an RDT, HRP-II based RDT appears as an important tool for detecting placental *P. falciparum* infection in women during pregnancy in Cameroon, since the standard diagnostic method used in this study is not recommended during pregnancy.

## Abbreviations

pLDH: *Plasmodium* lactate dehydrogenase
HRP-II: histidine-rich protein-II
RDT: rapid diagnostic test
ACT: artemisinin-based combination therapy
IVS: intervillous space
PTIS: placenta tissue impression smear
Pbs: peripheral blood smear
CSA: chondroitin sulfate A
WHO: World Health Organization
IPT: intermittent preventive treatment
Hb: haemoglobin
PPV: positive predictive value
NPV: negative predictive value
LLINs: long-lasting insecticidal net
